# Supplementary material for: Effect of Metabolic Adaptation by Voluntary Running Wheel Activity and Aldosterone Inhibition on Renal Function in Female Spontaneously Hypertensive Rats
Source: Cells. 2022 Dec 7;11(24):3954. doi: 10.3390/cells11243954 (PMC9777552; doi:10.3390/cells11243954)
Supplement: Supplementary file 1 [file cells-11-03954-s001.zip › cells-2037766-supplementary.pdf]

Fig. Suppl. 1: Study Protocol

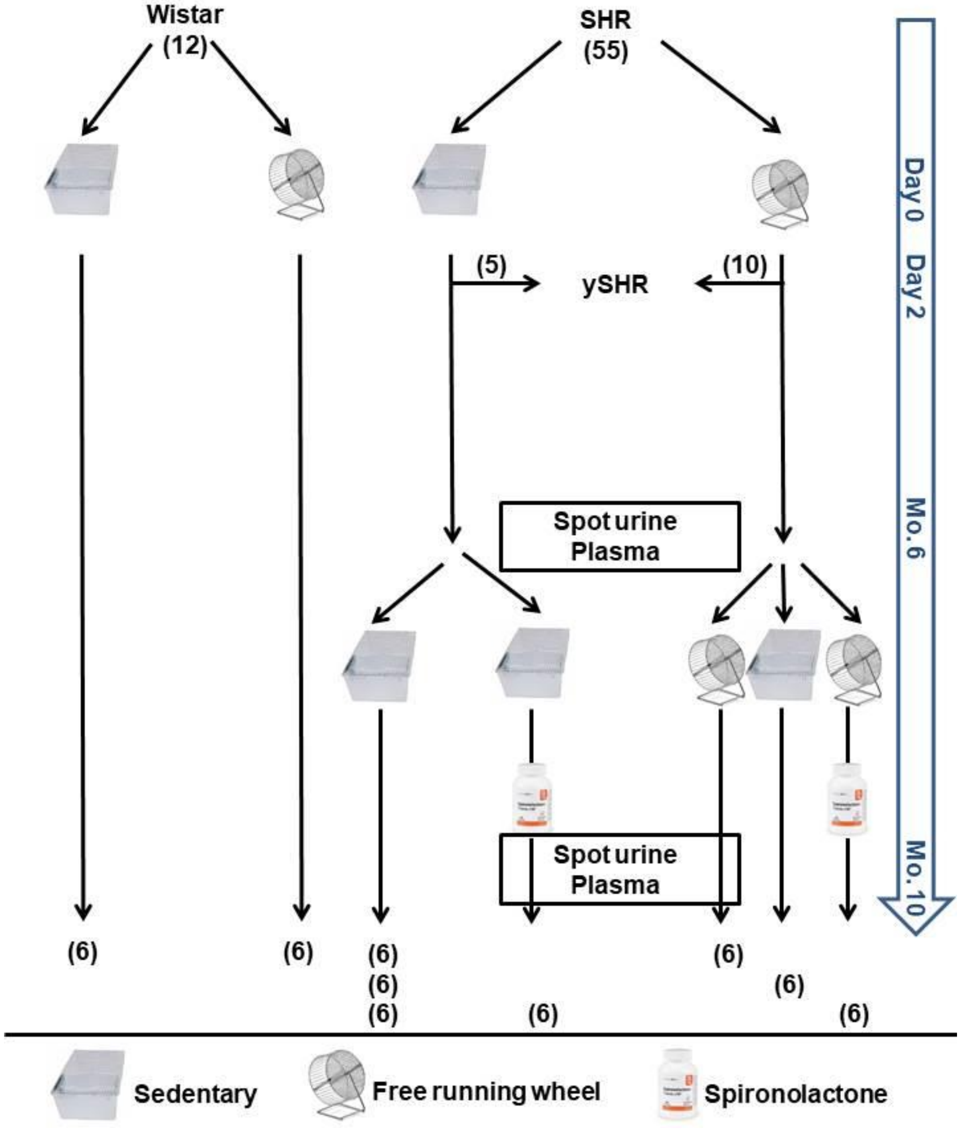

Table S1: Primer Sequences used for RT-PCR analysis

Gene	Forward	Reverse
ARG2	TGAGGAGCAGCGTCTCCCGT	GCTTCTCGGATGGCGGCTGG
B2M	GCCGTCGTGCTTGCCATTC	CTGAGGTGGGTGGAACTGAGAC
HAVCR1	CTTTGAGGAAGCCGCAGAGA	ATGTTGTCTTCAGCTCGGGG
LDLR	CTGGCGGCTGAGGAACATTA	ATCCTCCAGGCTGACCATCT
ODC	GAAGATGAGTCAAACGAGCA	AGTAGATGTTTGGCCTCTGG
PCSK9	TTGAACAAACTGCCCATCGC	CCCAACAGGTCACTGCTCAT
REN	GGAGGATGCCTCTCTGGGCACT	GATTTCCCGGACCGAGGGCA
